# Comment on “Cognitive performance protects against Alzheimer’s disease independently of educational attainment and intelligence” by Hu et al.

**DOI:** 10.1038/s41380-023-02374-8

**Published:** 2023-12-25

**Authors:** Charley Xia, W. David Hill

**Affiliations:** 1 Lothian Birth Cohort Studies, University of Edinburgh, 7 George Square, Edinburgh, EH8 9JZ UK; 2 School of Philosophy, Psychology and Language Sciences, Department of Psychology, University of Edinburgh, 7 George Square, Edinburgh, EH8 9JZ UK

**Keywords:** Biomarkers, Psychology

## To the Editor:

Alzheimer’s disease is the most common form of dementia (50–70% of cases) [1] and one of the leading causes of death in England and Wales [2]. The importance of developing strategies aiming to reduce the incidence of Alzheimer’s disease through modifiable risk factors is underscored by the inability of current treatments to reverse or delay disease progression.

Previous work using genome-wide association summary (GWAS) test statistics and multivariable Mendelian randomisation identified that a higher level of educational attainment and a higher level of intelligence are likely causal factors in Alzheimer’s disease, with intelligence affording a protective effect independent from that of educational attainment [3]. Published in this journal, Hu et al. [4] examined the effects of another variable, viz. “cognitive performance”. Again using GWAS summary statistics, they reported that a 1 SD increase in cognitive performance caused a 0.907 SD (95% CI = 0.877–0.938) increase in intelligence; moreover, a 1 SD increase in intelligence resulted in a 0.957 SD (95% CI = 0.937–0.978) increase in their so-called cognitive performance. Importantly, this cognitive performance also had a protective effect against Alzheimer’s disease that was independent of both education and intelligence [4]. Furthermore, Hu et al. [4] asserted that, whereas intelligence is largely fixed in early life, cognitive performance can be improved by education, exercise, and maintaining an active lifestyle; therefore, designing appropriate prevention strategies to increase cognitive performance might have clinical and public health implications by contributing to the reduction of Alzheimer’s disease.

Next, by carefully describing the sources of the GWAS summary statistics used for ‘intelligence’ and ‘cognitive performance’ in the Hu et al. [4] report, we ask whether these constructs are separable.

The instrumental variable used by Hu et al. [4] for ‘cognitive performance’ (*n* = 257,841) was obtained using GWAS summary statistics from a report by Lee at al. [5]. The variable was derived using 222,543 participants who undertook the verbal numerical reasoning test (also known as the fluid intelligence test) in the UK Biobank sample (UK Biobank data field 20016 for in-person assessments and data field 20191 for online assessment) and 35,298 participants of the COGENT consortium where the phenotype measured was the first unrotated principal component of performance on at least three neuropsychological tests or at least two IQ-test subscales. These data were then meta-analysed by Lee et al. [5] using Multi-Trait Analysis of GWAS (MTAG) [6] to capture the genetically correlated variance from three other cognitive performance related traits (educational attainment, highest mathematics qualification, and self-rated math ability). When cognitive performance was used as an outcome by Hu et al. [4], only the meta-analysis of UK Biobank and COGENT were used due to data availability.

When ‘intelligence’ was used as an outcome by Hu et al. [4], the data reported by Sniekers et al. [7] were used. These data contain 78,308 individuals of which 54,119 (a subset of both the participants used in Savage et al. [8] and Lee et al. [5]) were participants of UK Biobank who took the same verbal numerical reasoning test used in the cognitive performance phenotype derived by Lee et al. [5]. However, Sniekers et al. [7] included a measure of socio-economic status (SES, The Townsend Score) as an additional covariate that was not used in the Lee et al. [5] analysis.

When intelligence was used as an exposure by Hu et al. [4], 242 independent SNPs from the GWAS by Savage et al. [8] were used (*n* = 269,867). The GWAS on intelligence that was conducted by Savage et al. [8] utilised 195,653 participants who undertook the verbal numerical reasoning test in UK Biobank and so form a highly overlapping subset of participants that were in the Lee et al. [5] cognitive performance data set. Again, in this analysis by Savage et al. [8], socio-economic status was added as a covariate. This intelligence GWAS also used the same 35,298 participants of COGENT who completed the same tests as those used in the ‘cognitive performance’ phenotype derived by Lee et al. [5]. Savage et al. [8] also included an additional 35,993 participants sourced from independent epidemiological cohorts of European ancestry in which the phenotype was measured using various neurocognitive tests, primarily measuring fluid domains of cognitive functioning (See Table 1).

**Table 1:** Showing the cohorts used to derive the genetic instruments and outcomes for ‘cognitive performance’ and ‘intelligence’ from Hu et al. [4].

‘Cognitive performance’ (Lee et al. [5])			‘Intelligence’ (Savage et al. [8])			‘Intelligence’ (Sniekers et al. [7])		
Cohorts	*N*	Phenotype	Cohorts	*N*	Phenotype	Cohorts	*N*	Phenotype
UK Biobank	222,543	Verbal numerical reasoning (fluid intelligence)	UK Biobank	195,653	Verbal numerical reasoning (fluid intelligence using SES as an additional covariate)	UK Biobank	54,119	Verbal numerical reasoning (fluid intelligence using SES as an additional covariate)
COGENT	35,289	A g-factor was derived in each of the 35 cohorts of COGENT. The g-factor was formed in each cohort from at least two tests and was determined using the first unrotated component extracted from a principal components analysis of individual test scores.	COGENT	35,289	A g-factor was derived in each of the 35 cohorts of COGENT. The g-factor was formed in each cohort from at least two tests and was determined using the first unrotated component extracted from a principal components analysis of individual test scores.			
			Rotterdam Study	6182	g-factor	LBC1921	464	Moray House Test No.12
23andMe + Lee et al.	1,131,881	Educational attainment (meta-analysed using MTAG)	Generation R Study	1929	IQ score	LBC1936	947	Moray House Test No.12
23andMe	430,445	Highest Mathematics qualification (meta-analysed using MTAG)	Swedish Twin Registry	3215	g-factor	Brisbane Adolescent Twin Study	1752	IQ scores derived using Multi-dimensional Aptitude Battery
23andMe	564,698	Self-Rated Mathematics ability (meta-analysed using MTAG)	Spit for Science	2818	Scholastic Aptitude Test	Western Australia Pregnancy Cohort	2825	g-factor
			High IQ Health and retirement study	9410	High IQ case control study	Twins Early Development	2825	g-factor
			Twins Early Development	3414	Arithmetic mean of four tests.	Erasmus Rucphen Family Study	1076	g-factor
			IMAGEN	1343	g-factor	Generation R Study	3701	Snijders-Ooman non-verbal Intelligence Test
			Danish Twin Registry	990	A composite score derived using multiple cognitive domains	The Harvard/Union Study	389	IQ score
			Brisbane longitudinal Twin Study (Adult)	2598	IQ scores derived using Multi-dimensional Aptitude Battery	The Minnesota Centre for Twin and Family Research Study	3367	Wechsler Adult Intelligence Scale-Revised and the Wechsler Intelligence Scale for Children Revised
			Brisbane longitudinal Twin Study (Children)	530	g-factor	Swedish Twin Registry	3215	g-factor
			Netherlands study of cognition, environment and genes	252	Wechsler Adult Intelligence Scale	ALSPAC Children	5517	Wechsler Intelligence Scale for Children III
			Genes for Good	5084	Verbal reasoning test			
			Swedish Twin Studies of Aging	1191	g-factor			

Importantly for deriving instrumental variables, the Lee et al. [5] data on cognitive performance were meta-analysed with highest mathematics qualification, self-rated mathematics ability, and educational attainment using MTAG to add only the genetically-correlated variance to cognitive performance in order to derive trait-specific (i.e., cognitive performance) associations. The outcome GWAS data sets consisted of Sniekers et al. [7] (as described above) and Lee et al. [5] using only UK Biobank and COGENT without employing the MTAG procedure.

In psychology—although it may be applied beyond that discipline—there is an error known as the jangle fallacy [9]. One commits this error if one assumes that two identical (or near-identical) constructs are different if they happen to have different names. Unfortunately, Hu et al. [4] appear to have committed the jangle fallacy error by using intelligence and cognitive performance as if they were different variables. In this specific case of the jangle fallacy error, two data sets were used to make differently-named variables even though they contained the same participants who undertook exactly the same cognitive tests. The jangle fallacy error would still have been committed if another data set measuring intelligence (such as Sniekers et al. [7]) had been used as an exposure. As described above, these data sets are not identical but they measure the same phenotype as may be seen by the cognitive tests that were used (i.e., cognitive performance in Hu et al. [4] was measured using a test of fluid intelligence, and COGENT [10] derived a g factor from tests such as fluid reasoning, the Wechsler Adult Intelligence Scale—Revised, the Wechsler Abbreviated Scale of Intelligence, the Wechsler Adult Intelligence Scale I, II, and III).

The slight differences identified by Hu et al. [4] in the relationship that Alzheimer’s disease has with ‘intelligence’ and ‘cognitive performance’ is attributable to: (1) the bulk of the ‘intelligence’ data sets used in their instruments being conditioned on SES (195,653 participants’ scores were corrected for SES out of a total of 269,867 participants), whereas their ‘cognitive performance’ data set was not; (2) differences in the number of unique participants (Savage et al. [8] included 35,993 participants drawn from non-UK Biobank cohorts whereas Lee et al. [5] included an additional 26,890 UK Biobank participants); (3) and the ‘cognitive performance’ data set being meta-analysed with additional traits that capture SES. In the Hu et al. [4] report, intelligence and cognitive performance are not two correlated, but separate traits; rather, they are two measures of the same trait. We make the small caveat that the genetic variance associated with both SES and intelligence is absent from one of Hu et al. variables (‘intelligence’), and the genetic variance shared with educational attainment (often used as a measure of SES), highest mathematics qualification, and self-rated mathematics ability is better captured in the second (‘cognitive performance’).
